# Predicting radiotherapy-induced xerostomia in head and neck cancer patients using day-to-day kinetics of radiomics features

**DOI:** 10.1016/j.phro.2022.10.004

**Published:** 2022-11-04

**Authors:** Thomas Berger, David J. Noble, Leila E.A. Shelley, Thomas McMullan, Amy Bates, Simon Thomas, Linda J. Carruthers, George Beckett, Aileen Duffton, Claire Paterson, Raj Jena, Duncan B. McLaren, Neil G. Burnet, William H. Nailon

**Affiliations:** a Department of Oncology Physics, Edinburgh Cancer Centre, Western General Hospital, Crewe Road South, Edinburgh EH4 2XU, UK; b The University of Cambridge, Department of Oncology, Cambridge Biomedical Campus, Hills Road, Cambridge CB2 0QQ, UK; c Department of Clinical Oncology, Edinburgh Cancer Centre, Western General Hospital, Crewe Road South, Edinburgh EH4 2XU, UK; d Department of Medical Physics and Clinical Engineering, Cambridge University Hospitals NHS Foundation Trust, Cambridge Biomedical Campus, Hills Road, Cambridge CB2 0QQ, UK; e Edinburgh Parallel Computing Centre, Bayes Centre, 47 Potterrow, Edinburgh EH8 9BT, UK; f Beatson West of Scotland Cancer Centre, Great Western Road, Glasgow G12 0YN, UK; g The Christie NHS Foundation Trust, Wilmslow Road, Manchester, M20 4BX, UK; h School of Engineering, the University of Edinburgh, the King’s Buildings, Mayfield Road, Edinburgh EH9 3JL, UK

**Keywords:** Head and neck cancer, Radiomics, Xerostomia, Parotid gland, Mega-voltage CT

## Abstract

**Background and purpose:**

The images acquired during radiotherapy for image-guidance purposes could be used to monitor patient-specific response to irradiation and improve treatment personalisation. We investigated whether the kinetics of radiomics features from daily mega-voltage CT image-guidance scans (MVCT) improve prediction of moderate-to-severe xerostomia compared to dose/volume parameters in radiotherapy of head-and-neck cancer (HNC).

**Materials and Methods:**

All included HNC patients (N = 117) received 30 or more fractions of radiotherapy with daily MVCTs. Radiomics features were calculated on the contra-lateral parotid glands of daily MVCTs. Their variations over time after each complete week of treatment were used to predict moderate-to-severe xerostomia (CTCAEv4.03 grade ≥ 2) at 6, 12 and 24 months post-radiotherapy. After dimensionality reduction, backward/forward selection was used to generate combinations of predictors.

Three types of logistic regression model were generated for each follow-up time: 1) a pre-treatment reference model using dose/volume parameters, 2) a combination of dose/volume and radiomics-based predictors, and 3) radiomics-based predictors. The models were internally validated by cross-validation and bootstrapping and their performance evaluated using Area Under the Curve (AUC) on separate training and testing sets.

**Results:**

Moderate-to-severe xerostomia was reported by 46 %, 33 % and 26 % of the patients at 6, 12 and 24 months respectively. The selected models using radiomics-based features extracted at or before mid-treatment outperformed the dose-based models with an AUC_train_/AUC_test_ of 0.70/0.69, 0.76/0.74, 0.86/0.86 at 6, 12 and 24 months, respectively.

**Conclusion:**

Our results suggest that radiomics features calculated on MVCTs from the first half of the radiotherapy course improve prediction of moderate-to-severe xerostomia in HNC patients compared to a dose-based pre-treatment model.

## Highlights

• Textural features on daily images outperformed dose/volume parameters.
• The best models could be used before or at mid-treatment for personalisation.
• Area under the curve of the best models at 6, 12 and 24 months was 0.69, 0.74 and 0.86.

## 1 Introduction

Radiotherapy is an effective primary treatment modality for head and neck cancer (HNC) that can nevertheless result in life-altering morbidity, such as dry mouth or xerostomia [1], [2], [3], [4], [5]. Several clinical trials are however focussing on its reduction [6], [7], [8], [9] in light of significant advances [10]. Adaptive radiotherapy (ART) is emerging as a technique to limit radiation dose to critical structures [11] and alleviate the incidence of xerostomia [12]. However, selecting patients that would benefit from adaptation is challenging [13] and the analysis of images acquired during radiotherapy may offer a solution to this problem.

The science of radiomics aims to find robust statistical relationships between imaging patterns and clinical outcomes [14] and is a promising approach for data-driven selection of patients for treatment adaptation. This is made possible by the wealth of images generated during the radiotherapy workflow [15], [16], [17], [18]. Indeed, radiomics has already been used with a variety of imaging modalities to improve prediction of xerostomia. Whilst promising, many of these studies analysed single-timepoint pre-treatment images [19], [20], [21], [22], [23], ignoring differences in individual kinetics of imaging features calculated during treatment. A complementary approach was investigated by Wu et al. and van Dijk et al. who analysed the differences of imaging features of the parotid glands calculated on kilo-voltage CT (kVCT) scans acquired at different times of the radiotherapy course and found them to be associated with xerostomia [24], [25], [26].

Image-guided radiotherapy is now accepted as standard of care for many tumour sites [27], [28], but in current workflows the guidance images are essentially discarded once patient position is optimised, and any further useful information contained therein is lost. There is a growing interest in deeper mining of these images acquired at intervals during a course of radiotherapy [29], [30] as changes in imaging features may provide information on the patient-specific response to treatment - a phenomenon entirely neglected by current standard outcome predictors. To date, the potential of cone-beam CT image-guidance (CBCT-IG) analysis for predicting outcomes including xerostomia has been demonstrated but a similar deep analysis of the guidance images from helical radiotherapy platforms is yet to be carried out.

Therefore, the major objectives of the present work were: 1) to investigate whether image analysis of the parotid glands on MVCTs can improve prediction of moderate-to-severe xerostomia compared to standard dose/volume parameters; and 2) to identify the radiotherapy fractions associated with the best xerostomia prediction and consequently indicate the potential for treatment adaptation.

## 2 Material and methods

### 2.1 Study design, patient cohort and endpoints.

Data for this study were collected as part of the VoxTox study (UKCRN:13716) [31], [32], which received approval from the National Research Ethics Service Committee East of England (13/EE/0008) in 2013. All patients in the cohort were treated using TomoTherapy HiArt machines (Accuray, Sunnyvale, CA, USA) [31], [33] that deliver helical intensity modulated radiotherapy at each fraction after the acquisition of daily mega-voltage CT image-guidance (MVCT) scans. In the VoxTox study, these daily MVCT images were extracted from vendor archive, converted to dicom format, anonymised, and curated for analysis [31], [32].

A total of 337 patients with HNC were recruited to the VoxTox study. Inclusion criteria for this sub-study called IMAGE-INE were as follows. Firstly, selected patients all had radical radiotherapy schedules delivered in 30 fractions or more between 2014 and 2017, corresponding to improved HU stability of the TomoTherapy machines [34]. Second, inclusion required a dataset comprising a planning CT (pCT), dose matrix, full Organ At Risk (OAR) structure sets and daily MVCT including the parotid glands. This left a final cohort of 117 patients available for radiomics analysis. Their demographic and treatment characteristics are listed in Table 1. Toxicity data collected as part of the VoxTox was used for this study, with Common Terminology Criteria for Adverse Events (CTCAEv4.03) assessments [32], [33] at months 6, 12 and 24 after completion of therapy.

**Table 1:** Patients demographic and treatment characteristics.

		6 months	12 months	24 months
	Number of patients	112	95	57
	Moderate-to-severe xerostomia	51 (46 %)	31 (33 %)	15 (26 %)
	Contra-lateral parotid mean dose median [Q1 – Q3] (Gy)	29.8 [17.6–35.6]	29.6 [16.1–35.8]	25.1 [10.0–32.1]
	Age median [Q1 – Q3] (years)	58.5 [52–64.5]	59 [53–65]	58 [51–64.25]
Disease primary site	oropharynx	79	65	32
	Unknown primary	6	6	4
	oral cavity	9	8	8
	unspecified	9	8	5
	larynx	5	4	4
	maxilla	4	4	4
Dose prescription	60 Gy	22	20	18
	65 Gy	87	72	38
	70 Gy	3	3	1
Chemotherapy	None	34	28	23
	cetuximab	11	9	4
	cisplatin	67	58	30
tumour stageTNM (AJCC) 7	T0-2	77	64	40
	T3-4	35	31	17
nodal statusTNM (AJCC) 7	N0-1	41	34	20
	N2-3	71	61	37

The chosen endpoint was moderate-to-severe xerostomia post-radiotherapy, as defined by a toxicity score ≥ 2. In the selected cohort, toxicity scores were available for 112, 95 and 57 patients at 6, 12 and 24 months, respectively. In this study, the parotid gland with the minimum mean dose was considered as contra-lateral.

### 2.2 Treatment details, image analysis and feature extraction

Treatment details for the patients in this study are as previously published [33]. Target-related and OAR contours relevant for radiotherapy were delineated on each slice of the pCT of each patient by site-expert HNC radiation oncologists [33] according to established protocols and contouring atlases [35], [36], [37]. Parotid gland contours were propagated from the pCT to the daily MVCTs using the Elastix deformable image registration (DIR) tool [33], [38]. Clinical evaluation of the resulting contours was performed independently by clinicians [33], [39]. The training of the DIR model was performed by computing Dice Similarity Coefficient (DSC), Mean Surface Distance as well as Volume Difference values to adjust the parameters. The performance of the selected DIR model was similar to the one obtained by HNC specialists as mean DSC scores of 0.80 and 0.82 for Elastix and inter-observer variability were found, respectively, on a blind testing set. The parotid contours at fraction 1 and 30 of two patients that significantly changed morphologically during treatment are shown in Supplementary Material A. Also, slices with high HU dental implants were disregarded to avoid analysing lower quality voxels [40], [41] due to beam hardening.

In this study, patients alternated between two radiotherapy machines that had intrinsic HU calibration differences [34]. Therefore, the MVCTs were normalised by shifting the HU of the organs of interest by the differences observed monthly between the two machines. All the steps of the present study, including image and statistical analysis were performed using MatLab (Mathworks, Natick, MA, USA) software.

The principal mechanism underpinning radiomics analysis is to calculate features that correlate with the underlying pathology. The hypothesis investigated here is that patients experiencing xerostomia have specific variations in MVCT image texture or, in other words, a unique radiomics signature. A total of 123 radiomics features, listed in Supplementary Material B and defined according to the Image Biomarker Standardisation Initiative (IBSI) [42], [43], were calculated on the daily contra-lateral parotid gland contours from the MVCTs. Previous publications have shown their superiority compared to the ipsi-lateral glands for xerostomia prediction [25], [44], [45]. The feature extraction scripts were benchmarked using reference values provided on the IBSI website [43].

### 2.3 Data analysis and outcome modelling

**Fig. 1:**
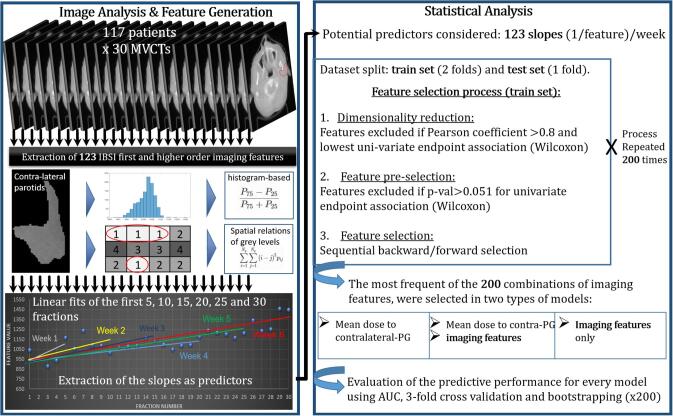
Diagram illustrating the main methodological steps from radiomics features extraction to development and evaluation of predictive models.

The main analysis steps are illustrated in Fig. 1. After the extraction phase, feature values were plotted against the fraction number. Linear regressions were performed between fraction one and the fractions that were multiples of five, thus corresponding to complete weeks of treatment. The slopes of the regressions show the rate of change, or kinetics of the features, and were extracted as potential predictors of xerostomia. Also, mean dose to the contra-lateral parotid was extracted from the pCT as a predictor. Other clinical factors were considered especially baseline toxicity scores, as it was found in previous studies [44], [46] to be an important predictor of late xerostomia. For this cohort, however, the added value of this predictor was negligible or unfavourable for later endpoints and was therefore omitted. The predictors’ values were then standardised by subtracting the average across all patients and dividing by the standard deviation.

To standardise the scientific quality and reporting of radiomics studies the TRIPOD (Transparent Reporting of a multivariable prediction model for Individual Prognosis Or Diagnosis) initiative recommends following a number of reporting guidelines [47]. Supplementary Material C contains a checklist showing our adherence to TRIPOD. In line with this process, an estimate of the predictive performance of our statistical models was obtained using a re-sampling procedure that included cross-validation and bootstrapping for the purpose of avoiding overfitting.

Pre-treatment reference models that used mean dose to the contra-lateral parotid glands as predictors were considered as the standard for comparison. To quantify the added value of imaging features, two additional models were created by combining:

1/Radiomics-based predictors.
2/Radiomics-based predictors with mean dose.

To visualise the potential for treatment adaptation, one radiomics-based model was generated at the end of every week after the start of treatment. This process was repeated for every follow-up time.

Several precautionary measures detailed in Fig. 1 were implemented in the generation of each model to maximise their robustness. First, the patients were randomly split into three folds with similar proportions of patients with toxicity. A train set composed of two folds was used to carry out the selection process which consisted of three phases detailed below.

The first step was to reduce dimensionality by excluding radiomics-based predictors with a Pearson correlation coefficient exceeding 0.8 and the lower uni-variable association (Wilcoxon test) with the endpoint. Then, all predictors associated with a univariate (Wilcoxon test) p-value >0.051 were also excluded from further analysis. Finally, sequential backward/forward selection was used to choose among the remaining predictors. This three-step selection process was applied 200 times to two-thirds of the patients and for every model generation. In this way, 200 combinations of predictors were formed at each week and each follow-up time. The most frequently generated combinations, hence considered the most robust, were selected for further analysis and their predictive power evaluated.

The three types of logistic regression models were fitted to predict moderate-to-severe xerostomia at 6, 12 and 24 months after radiotherapy. Model performance was evaluated based on its ability to correctly classify patients using Area Under the Curve (AUC), specificity, sensitivity and accuracy. To determine the values of the latter three metrics, the cost of misclassifying a positive class (xerostomia patient) as a negative class was set to be three times higher than the cost of misclassifying a negative class as a positive class. This was motivated by clinical considerations, as adapting treatments to patients who would not suffer from xerostomia only represents an additional workload while failing to adapt treatments to patients who will suffer from xerostomia will result in a degraded quality of life. In addition, 3-fold cross-validation was used to internally validate the models. The aforementioned performance metrics were extracted on the training and testing sets, the folds were then rotated and the process repeated. Model robustness was also evaluated using bootstrapping by repeating 200 times the random allocation of patients into folds. Three models presented in detail in Table 2 and Supplementary Material D were then selected based on their AUC on the testing set (AUC_test_), provided that the radiomics-based predictors came from slopes calculated within the first three weeks of treatment. This ensured that the patients could benefit from treatment personalisation for at least the second half of the radiotherapy course. Their corresponding ROC curves and calibration plots are shown in Supplementary Material E and F.

**Table 2:** Selected models for xerostomia prediction at 6, 12 and 24 months and their associated performance. fx1_X indicates a slope extracted from a linear regression including fractions 1 to X. Confidence Interval [CI] is given by 5th and 95th percentiles.

	models	fraction adaptation	AUC train [CI]	AUC test [CI]
6 m	Mean Dose + information correlation 1 GLCM 2D fx1_5	5	0.70[0.70–0.71]	0.69[0.65–0.71]
	information correlation 1 GLCM 2D fx1_5	5	0.67[0.66–0.67]	0.67[0.65–0.68]
	Mean Dose	0	0.64[0.64–0.65]	0.64[0.63–0.66]
12 m	Mean Dose + Q1-Q3 range HU 3D fx1_10 + min hist grad int HU 3D fx1_10	10	0.76[0.75–0.77]	0.73[0.69–0.76]
	Q1-Q3 range HU 3D fx1_10 + min hist grad int HU 3D fx1_10	10	0.76[0.75–0.76]	0.74[0.71–0.76]
	Mean Dose	0	0.61[0.61–0.62]	0.61[0.59–0.63]
24 m	Mean Dose + Normalised Grey Level Non Uniformity GLRLM 2D fx1_15	15	0.85[0.84–0.86]	0.82[0.75–0.86]
	Normalised Grey Level Non Uniformity GLRLM 2D fx1_15	15	0.86[0.85–0.86]	0.86[0.83–0.88]
	Mean Dose	0	0.59[0.58–0.60]	0.59[0.54–0.61]

Finally, to evaluate the added value that radiomics features have for xerostomia prediction compared with parotid volume variations, which has been shown previously to be associated with xerostomia symptoms, correlation between these variables on our dataset was assessed and presented in Supplementary Material G.

## 3 Results

Of the patients with toxicity available, 51 (46 %) reported moderate-to-severe xerostomia at 6 months, 31 (33 %) at 12 months and 15 (26 %) at 24 months.

**Fig. 2:**
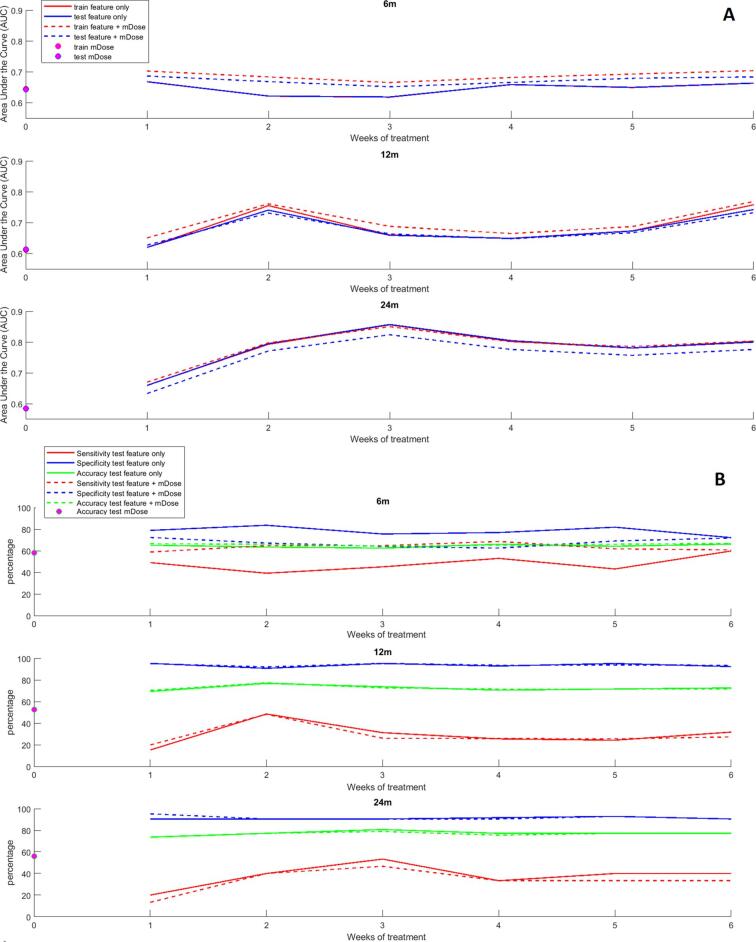
Plot showing training and testing AUC, sensitivity, specificity and accuracy, for the selected models of both types studied. Magenta rounds show the performance of models composed of dose/volume parameters. cPG = contra-lateral parotid gland; mDose = mean dose. (For interpretation of the references to colour in this figure legend, the reader is referred to the web version of this article.)

At the end of the selection process, the resulting combinations for modelling xerostomia development were systematically composed of one predictor chosen among the 123 slopes present at each complete week after the start of treatment, with the exception of week 2 and 6 for prediction of xerostomia at 12 months where there were two. The frequency of selection of the features for the models presented in Table 2 is shown in Supplementary Material H. As shown in Fig. 2, the reference models composed of mean dose to the parotid yielded an AUC_train_/AUC_test_ of 0.64/0.64 at 6 months, 0.61/0.61 at 12 months and 0.59/0.59 at 24 months. As shown in detail in Table 2, the addition of one single feature to the models considerably improved prediction especially for longer follow-up time and for the selected models this results in an AUC_train_/AUC_test_ of 0.70/0.69 at 6 months and 0.76/0.73 at 12 months and 0.85/0.82 at 24 months. Models composed solely of radiomics features yielded AUC_train_/AUC_test_ of 0.67/0.67 at 6 months 0.76/0.74 at 12 months and 0.86/0.86 at 24 months. The maximum AUC_test_ could always be obtained for the two types of radiomics-based models within the first three weeks of treatment. For predicting xerostomia at 6 months, the addition of the mean dose to the radiomics-based model is improving the predictive performance for all six models (Fig. 2A and upper graph). However for predicting xerostomia at longer follow-up times, the gains of adding the dose information are smaller or negative (for 24 months) because of the slight overfitting it caused.

## 4 Discussion

On the cohort analysed composed of 117 patients with HNC, models that included radiomics features of the parotid glands from daily MVCTs were found to outperform dose-based models in predicting moderate-to-severe xerostomia. Furthermore, the best performing models were associated with an AUC_test_ of 0.69, 0.74 and 0.86 for predicting toxicity at 6, 12 and 24 months respectively and could all be used for personalisation at mid-treatment.

The predictive performance of models only using the mean dose to the contra-lateral parotid gland was found to decrease for longer follow-up times with an AUC_test._ of 0.64, 0.61 and 0.59 for predicting xerostomia at 6, 12 and 24 months. Interestingly, the trend is opposite and the performance higher in the selected models only using radiomics-based predictors, with an AUC_test_ of 0.67, 0.76 and 0.86 for predicting xerostomia at 6, 12 and 24 months. Therefore, the inclusion of textural features in the models improves xerostomia prediction on our cohort compared to models that only use dose/volume parameters. A potential explanation for these results is that the kinetics of radiomics features may provide information on the patient-specific response to irradiation. The performance achieved is close to that of Rosen et al., who obtained an AUC of 0.77 by combining imaging features calculated on CBCT scans with clinical and dose/volume parameters in 105 patients [30]. In a similar study, van Dijk et al. found an AUC_test_ of 0.93 by using geometric characteristics calculated on weekly diagnostic quality CTs of 68 patients and combining them with parotid gland mean dose and baseline xerostomia scores [25]. However, a significant difference in our study design compared to the common approaches in the literature is the use of daily time-of-treatment imaging which enabled us to determine the kinetics of radiomics features with a quotidian resolution. Also, the combination of dose and radiomics information clearly improved on our cohort the predictive performance at 6 months. However the gains decreased for longer follow-up times and even became negative when predicting xerostomia at 24 months because of the slight overfitting caused. This study also supports the conclusions of Gu et al. who found MVCTs to have potential for building predictive models [48].

The predictors included in the selected models are from week 1, 2 and 3 for predicting xerostomia at 6, 12 and 24 months, respectively. This shift towards the end of the treatment course for later xerostomia symptoms which can be visualised on Fig. 2, suggests that variations in radiomics features occurring early in the radiotherapy course are related to xerostomia symptoms at 6 months, but that prediction of long-term xerostomia requires longer time-intervals of the treatments to be analysed.

It is also interesting to note that, radiomics features from the first week of treatment provide key information for predicting xerostomia as, for the three follow-up times, models only using those performed similarly or better than dose alone. These results open up the potential for setting up early adaptation protocols that could benefit the significant proportion of patients now treated on helical radiotherapy platforms. ART allows for a decrease in toxicity of HNC patients by accounting for changes in both tumour and normal tissues [49], [50]. ART is labour and resource intensive, not all patients will benefit from this approach, however, selection methods based on patient-specific responses such as in this study show promise [51].

The previous studies that investigated the predictive power of variations of imaging features during radiotherapy did so using “delta-radiomics” [24], [25], [26]. Delta-radiomics involves looking at the absolute variation by a simple subtraction of the two values. In this work, thanks to the availability of daily images, we performed a linear regression of feature variations and extracted the slopes as predictors. Because of this extra step, potential random errors in images would, on average, be balanced out.

Whilst the results of our study are encouraging and may have relevant clinical implications, some inherent limitations must be acknowledged. In particular, although a large number of patients were initially recruited to this study, the number of patients with toxicity reported decreased over time after radiotherapy. As a result, a modest number of patients was analysed at 24 months. Another limitation of our study is that all the patients included were treated at a single centre. To establish the validity of our results, internal validation was carried out by testing model performance blindly. Despite these precautions, our results need to be externally validated. There are many steps involved in developing reliable radiomics-based models and, to date, no solution exists that could be adopted by multiple clinics to assist in optimal ART patient selection. As discussed by Zwanenburg et al., large-scale multi-institutional studies are required to fully realise the power of radiomics in this area and the IBSI is one such example [42].

In conclusion, models using textural features calculated on daily MVCTs outperformed models based on dose/volume parameters in predicting xerostomia. The best performing models were associated with an AUC_test_ of 0.69, 0.74 and 0.86 for predicting toxicity at 6, 12 and 24 months respectively and could all be used before or at mid-treatment for personalising treatments to individual needs. Provided these findings are externally validated, radiomics features derived from MVCT image-guidance scans could be incorporated into established NTCP models and contribute to patient selection for adaptation of HNC patients.

## Declaration of Competing Interest

The authors declare the following financial interests/personal relationships which may be considered as potential competing interests: NB, LJC, RJ, AB, WHN, DJN, GB, LEAS, TB, DM, TM report grants from Chief Scientist Office (CSO) Scotland grant (TCS/17/26 - CSO Award), during the conduct of the study. The authors alone are responsible for the content and writing of the paper. LJC reports personal fees from BrainLAB - Novalis Certified, outside the submitted work; RJ reports personal fees from Microsoft, outside the submitted work; DJN reports grants from Cancer Research UK Clinical Research Fellowship (Ref: C20/A20917), grants from Cancer Research UK Programme Grant (Ref: C8857/A13405), during the conduct of the study; LEAS reports grants from University of Cambridge WD Armstrong Trust, outside the submitted work; AD, CP, and ST have nothing to disclose.
