# Supplementary material for: Predicting radiotherapy-induced xerostomia in head and neck cancer patients using day-to-day kinetics of radiomics features
Source: Phys Imaging Radiat Oncol. 2022 Nov 4;24:95–101. doi: 10.1016/j.phro.2022.10.004 (PMC9647222; doi:10.1016/j.phro.2022.10.004)
Supplement: Supplementary data 1 [file mmc1.docx]

**Supplementary Material**

**A:** Example of parotid contours at fraction 1 and 30 for two patients that changed morphologically during treatment. The colours indicate different regions of the gland and the whole structure is defined by both regions regardless of the colour.

**B:** list of the 123 radiomics features derived by image analysis. The Image Biomarker Standardisation Initiative ([https://ibsi.readthedocs.io/en/latest/](about:blank)) was followed for the nomenclature as well as for benchmarking the feature calculations.

**C:** TRIPOD (Transparent Reporting of a multivariable prediction model for Individual Prognosis Or Diagnosis) checklist

**D:** Details of the selected models

**E:** ROC curves of the radiomics-based models presented in Table 2

**F**: Calibration plots of the radiomics-based models presented in Table 2

**G:** Predictive performance of parotid glands volume variations and their correlation with the selected radiomics features.

**H:** Number of selections per radiomic feature for the selected models out of 200 iterations. The total may exceed 200 as combinations of several predictors could be selected.

**A:** Example of parotid contours at fraction 1 and 30 for two patients that changed morphologically during treatment. The colours indicate different regions of the gland and the whole structure is defined by both regions regardless of the colour.


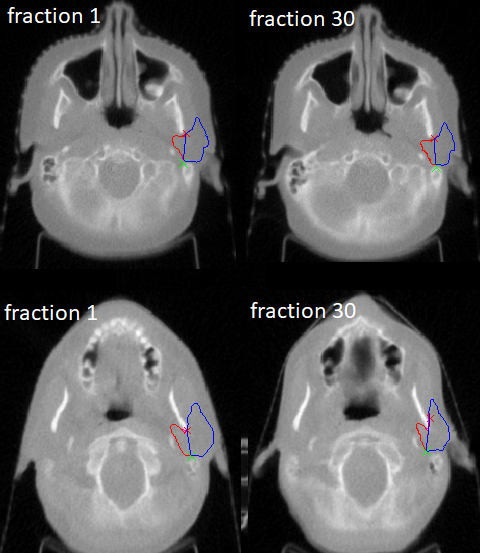


**B:** list of the 123 radiomics features derived by image analysis. The Image Biomarker Standardisation Initiative ([https://ibsi.readthedocs.io/en/latest/](about:blank)) was followed for the nomenclature as well as for benchmarking the feature calculations.

| number | list_of_all_features |
| --- | --- |
| 1 | mean_HU_3D |
| 2 | variance_HU_3D |
| 3 | skewness_HU_3D |
| 4 | kurtosis_HU_3D |
| 5 | min_int_HU_3D |
| 6 | P_10_HU_3D |
| 7 | median_int_HU_3D |
| 8 | P_90_HU_3D |
| 9 | P_97_5_HU_3D |
| 10 | max_int_HU_3D |
| 11 | Q1_Q3_range_HU_3D |
| 12 | mean_abs_dev_HU_3D |
| 13 | rob_mean_abs_dev_HU_3D |
| 14 | med_abs_dev_HU_3D |
| 15 | coef_of_variation_HU_3D |
| 16 | Q1Q3_coef_dispersion_HU_3D |
| 17 | energy_HU_3D |
| 18 | RMS_HU_3D |
| 19 | hist_mode_HU_3D |
| 20 | entropy_HU_3D |
| 21 | uniformity_HU_3D |
| 22 | max_hist_grad_HU_3D |
| 23 | max_hist_grad_int_HU_3D |
| 24 | min_hist_grad_HU_3D |
| 25 | min_hist_grad_int_HU_3D |
| 26 | vol_fx_at_10prct_int_HU_3D |
| 27 | vol_fx_at_90prct_int_HU_3D |
| 28 | int_at_10prct_vol_HU_3D |
| 29 | int_at_90prct_vol_HU_3D |
| 30 | joint_max_GLCM_2D |
| 31 | joint_average_GLCM_2D |
| 32 | joint_variance_GLCM_2D |
| 33 | joint_entropy_GLCM_2D |
| 34 | difference_average_GLCM_2D |
| 35 | difference_variance_GLCM_2D |
| 36 | difference_entropy_GLCM_2D |
| 37 | sum_average_GLCM_2D |
| 38 | sum_variance_GLCM_2D |
| 39 | sum_entropy_GLCM_2D |
| 40 | angular_second_moment_GLCM_2D |
| 41 | contrast_GLCM_2D |
| 42 | inverse_difference_GLCM_2D |
| 43 | normalised_inverse_difference_GLCM_2D |
| 44 | inverse_difference_moment_GLCM_2D |
| 45 | normalised_inverse_difference_moment_GLCM_2D |
| 46 | inverse_variance_GLCM_2D |
| 47 | correlation_GLCM_2D |
| 48 | autocorrelation_GLCM_2D |
| 49 | cluster_tendency_GLCM_2D |
| 50 | cluster_shade_GLCM_2D |
| 51 | cluster_prominence_GLCM_2D |
| 52 | information_correlation_1_GLCM_2D |
| 53 | information_correlation_2_GLCM_2D |
| 54 | Short_Run_Emphasis_GLRLM_2D |
| 55 | Long_Run_Emphasis_GLRLM_2D |
| 56 | Short_Run_High_Gray_Emphasis_GLRLM_2D |
| 57 | Low_Gray_Level_Run_Emphasis_GLRLM_2D |
| 58 | High_Gray_Level_Run_Emphasis_GLRLM_2D |
| 59 | Short_Run_Low_Gray_Emphasis_GLRLM_2D |
| 60 | Long_Run_Low_Gray_Emphasis_GLRLM_2D |
| 61 | Long_Run_High_Gray_Emphasis_GLRLM_2D |
| 62 | Grey_Level_Non_Uniformity_GLRLM_2D |
| 63 | Normalised_Grey_Level_Non_Uniformity_GLRLM_2D |
| 64 | Run_Length_Non_Uniformity_GLRLM_2D |
| 65 | Normalised_Run_Length_Non_Uniformity_GLRLM_2D |
| 66 | Run_Percentage_GLRLM_2D |
| 67 | Grey_Level_Variance_GLRLM_2D |
| 68 | Run_Length_Variance_GLRLM_2D |
| 69 | Run_Entropy_GLRLM_2D |
| 70 | Small_Distance_Emphasis_GLDZM_2D |
| 71 | Large_Distance_Emphasis_GLDZM_2D |
| 72 | Low_Grey_Level_Zone_Emphasis_GLDZM_2D |
| 73 | High_Grey_Level_Zone_Emphasis_GLDZM_2D |
| 74 | Small_Distance_Low_Grey_Level_Emphasis_GLDZM_2D |
| 75 | Small_Distance_High_Grey_Level_Emphasis_GLDZM_2D |
| 76 | Large_Distance_Low_Grey_Level_Emphasis_GLDZM_2D |
| 77 | Large_Distance_High_Grey_Level_Emphasis_GLDZM_2D |
| 78 | Grey_Level_Non_Uniformity_GLDZM_2D |
| 79 | Normalised_Grey_Level_Non_Uniformity_GLDZM_2D |
| 80 | Zone_Distance_Non_Uniformity_GLDZM_2D |
| 81 | Normalised_Zone_Distance_Non_Uniformity_GLDZM_2D |
| 82 | Zone_Percentage_GLDZM_2D |
| 83 | Grey_Level_Variance_GLDZM_2D |
| 84 | Zone_Distance_Variance_GLDZM_2D |
| 85 | Zone_Distance_Entropy_GLDZM_2D |
| 86 | Small_Zone_Emphasis_GLSZM_2D |
| 87 | Large_Zone_Emphasis_GLSZM_2D |
| 88 | Low_Grey_Level_Zone_Emphasis_GLSZM_2D |
| 89 | High_Grey_Level_Zone_Emphasis_GLSZM_2D |
| 90 | Small_Zone_Low_Grey_Level_Emphasis_GLSZM_2D |
| 91 | Small_Zone_High_Grey_Level_Emphasis_GLSZM_2D |
| 92 | Large_Zone_Low_Grey_Level_Emphasis_GLSZM_2D |
| 93 | Large_Zone_High_Grey_Level_Emphasis_GLSZM_2D |
| 94 | Grey_Level_Non_Uniformity_GLSZM_2D |
| 95 | Normalised_Grey_Level_Non_Uniformity_GLSZM_2D |
| 96 | Zone_Size_Non_Uniformity_GLSZM_2D |
| 97 | Normalised_Zone_Size_Non_Uniformity_GLSZM_2D |
| 98 | Zone_Percentage_GLSZM_2D |
| 99 | Grey_Level_Variance_GLSZM_2D |
| 100 | Zone_Size_Variance_GLSZM_2D |
| 101 | Zone_Size_Entropy_GLSZM_2D |
| 102 | Low_Dependence_Emphasis_NGLDM_2D |
| 103 | High_Dependence_Emphasis_NGLDM_2D |
| 104 | Low_Grey_Level_Count_Emphasis_NGLDM_2D |
| 105 | High_Grey_Level_Count_Emphasis_NGLDM_2D |
| 106 | Low_Dependence_Low_Grey_Level_Emphasis_NGLDM_2D |
| 107 | Low_Dependence_High_Grey_Level_Emphasis_NGLDM_2D |
| 108 | High_Dependence_Low_Grey_Level_Emphasis_NGLDM_2D |
| 109 | High_Dependence_High_Grey_Level_Emphasis_NGLDM_2D |
| 110 | Grey_Level_Non_Uniformity_NGLDM_2D |
| 111 | Normalised_Grey_Level_Non_Uniformity_NGLDM_2D |
| 112 | Dependence_Count_Non_Uniformity_NGLDM_2D |
| 113 | Normalised_Dependence_Count_Non_Uniformity_NGLDM_2D |
| 114 | Dependence_Count_Percentage_NGLDM_2D |
| 115 | Grey_Level_Variance_NGLDM_2D |
| 116 | Dependence_Count_Variance_NGLDM_2D |
| 117 | Dependence_Count_Entropy_NGLDM_2D |
| 118 | Dependence_Count_Energy_NGLDM_2D |
| 119 | Coarsness_NGTDM_2D |
| 120 | Contrast_NGTDM_2D |
| 121 | Busyness_NGTDM_2D |
| 122 | Complexity_NGTDM_2D |
| 123 | Strength_NGTDM_2D |

**C:** TRIPOD (Transparent Reporting of a multivariable prediction model for Individual Prognosis Or Diagnosis) checklist


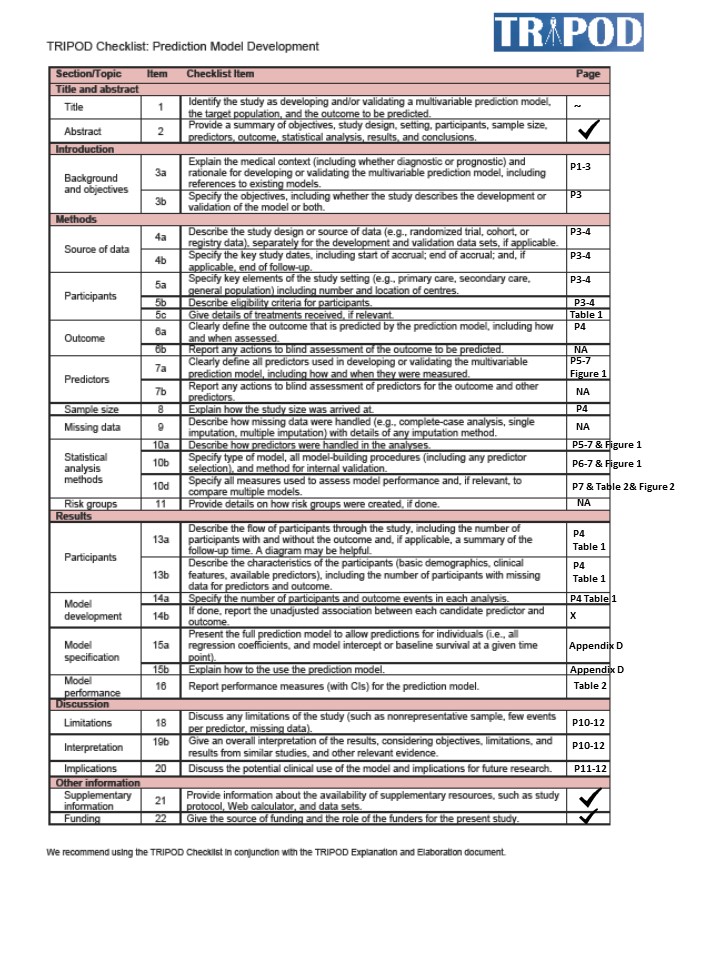


**D:** Details of the selected models


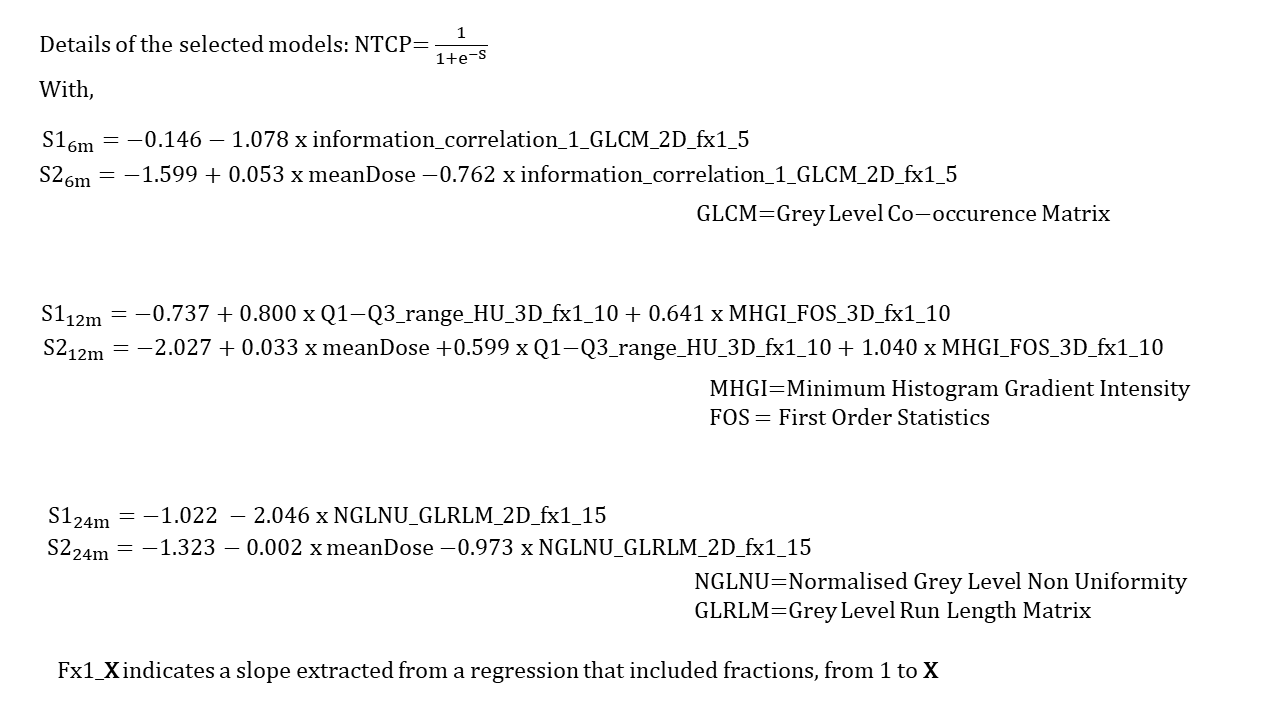


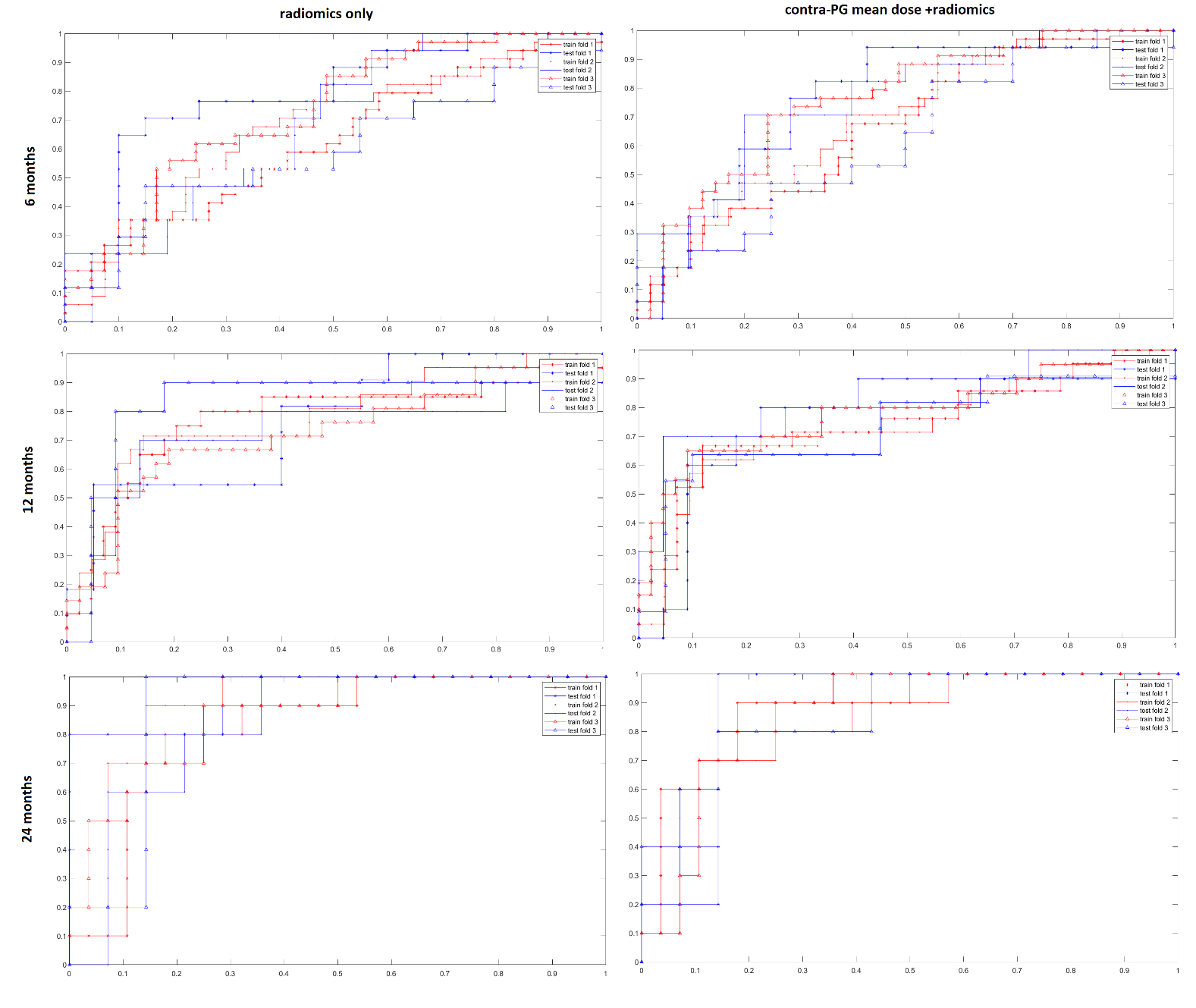
**E:** ROC curves of the radiomics-based models presented in Table 2

**F**: Calibration plots of the radiomics-based models presented in Table 2


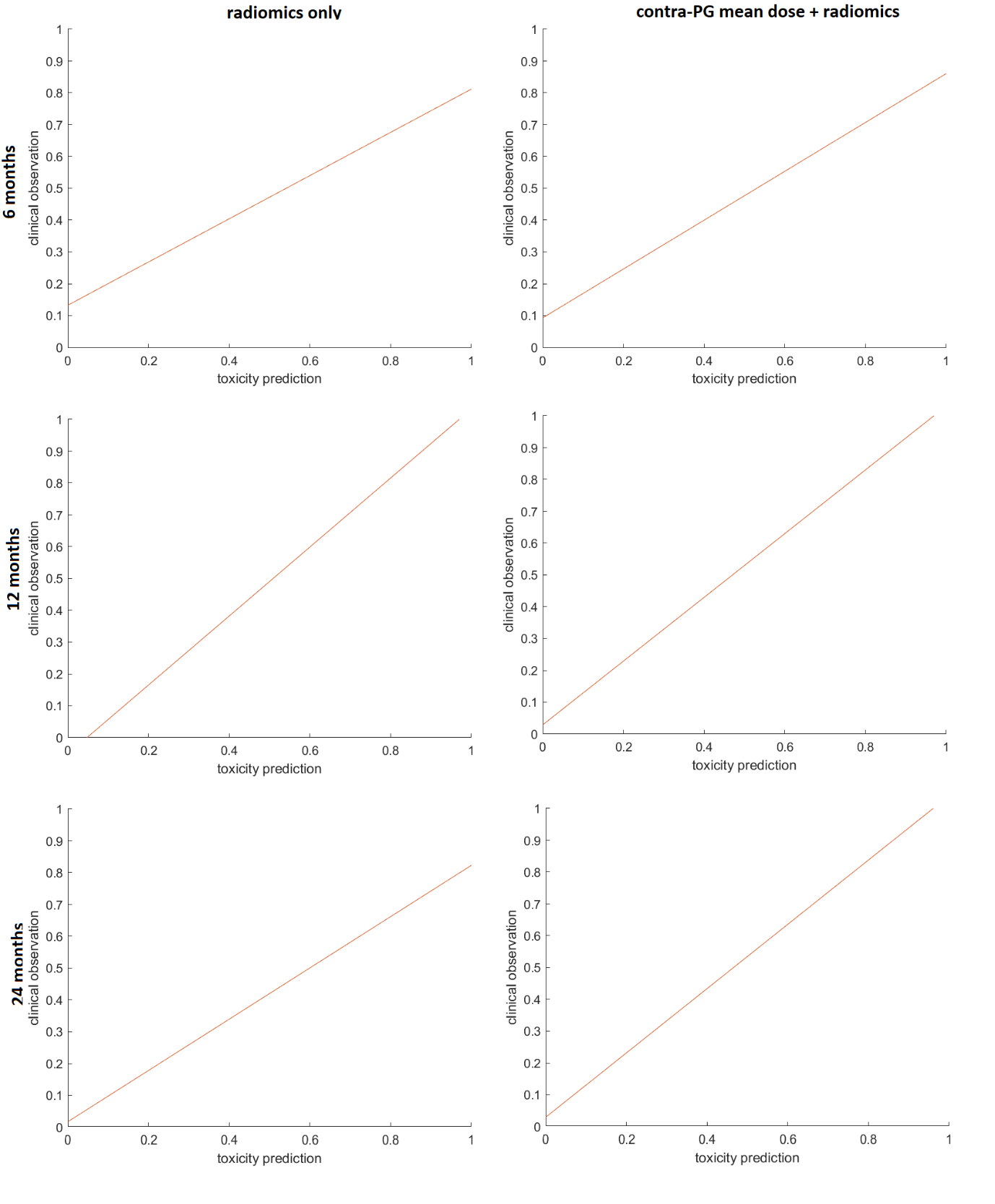


**G:** Predictive performance of parotid glands volume variations and their correlation with the selected radiomics features.

The volume of parotid glands at the start of treatment as well as volume variations during treatment have previously been shown to be associated with xerostomia symptoms.

In addition, the results of anterior studies indicated that radiomics features, in some cases, could be strongly correlated with the volume of the organ analysed. The presence of such a correlation would render radiomics analysis unnecessary as radiomics feature extraction is more complex, time- and work- intensive than a simple volume determination.

In order to investigate on our dataset whether parotid gland volume variations correlate with, or perform similarly to the radiomics features of the models selected in this study, we calculated for each patient the variations in volume (slopes) of the most voluminous slice composing the contra-lateral parotids for the 6 weeks of treatment.

Based on the results shown just below, the trend follows the commonly reported decrease in volume which becomes more stable by the end of the radiotherapy course.


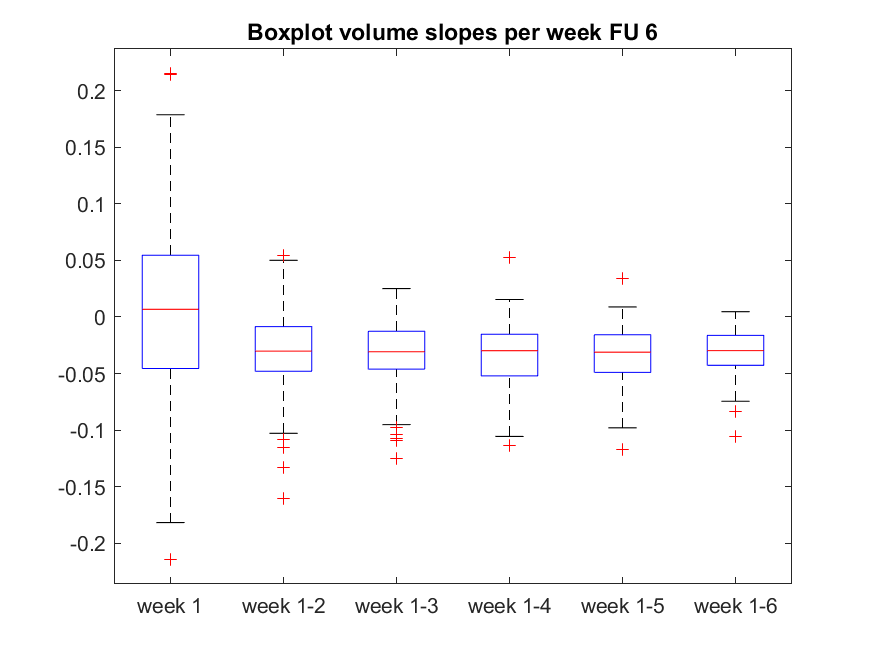


In a second phase we investigated how the radiomics features composing the selected models (Table 2) correlate with the volume variations (slope) observed using Pearson correlation and plotted the results in the correlation matrix below:


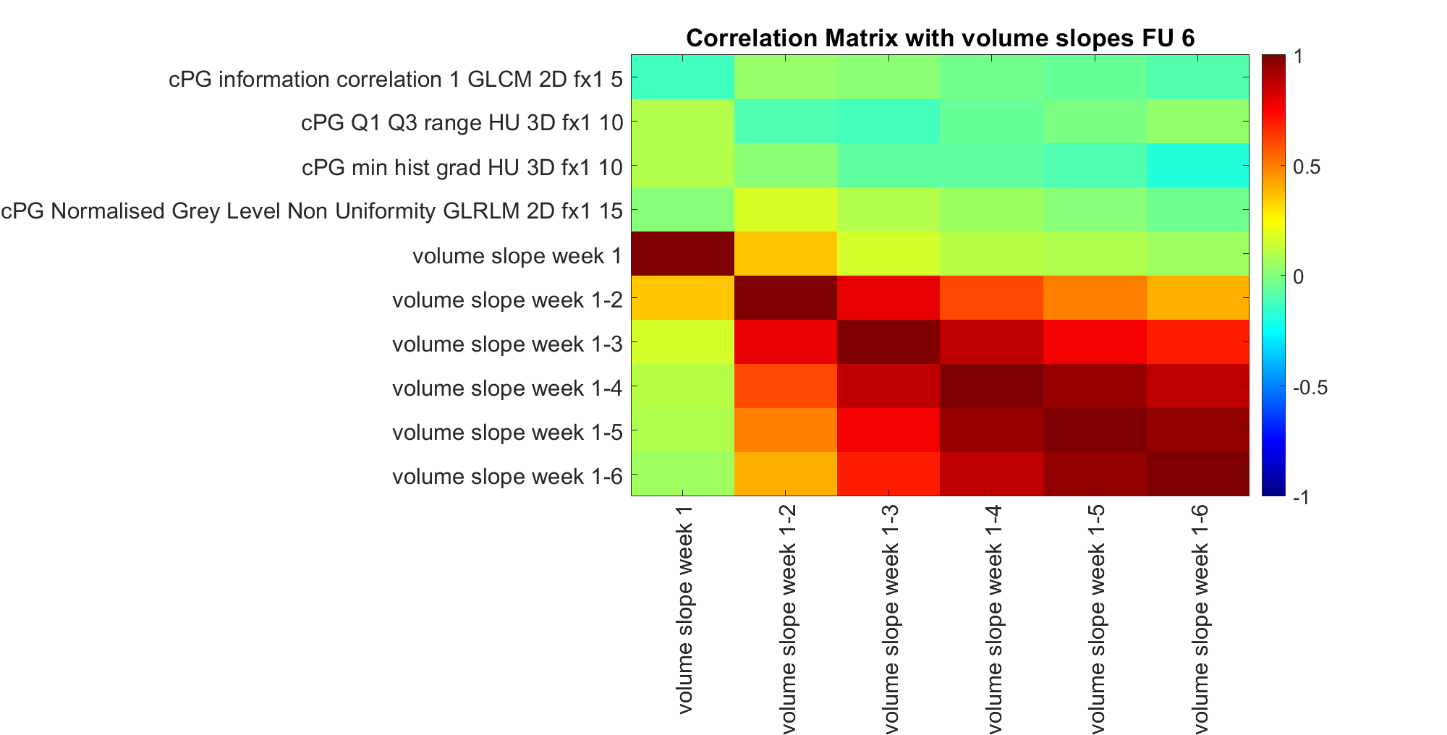


The volume variations of consecutive weeks are well correlated, especially from week 2 onwards, with an absolute Pearson correlation coefficient ranging from 0.40 to 0.96, as indicated by the red rectangular area at the bottom right of the above figure. However, the selected radiomics features correlate relatively poorly with volume variations with absolute correlation coefficients ranging from 0.01 to 0.18. This demonstrates clearly that the selected features are not surrogate of volume variation.

Finally, we investigated whether volume variation could be used to predict xerostomia, as reported in other studies, and could perform similarly or better than the radiomics-based selected models.

For that, we used the whole cohorts available at 6, 12 and 24 months and for each used respectively the parotid volume variation at weeks 1, 2 and 3 (identical to the times at which prediction can be achieved using the selected models of Table 2) to predict xerostomia at 6, 12 and 24 months. The AUCs obtained are 0.55, 0.55, 0.64. When volume variations at week 5 was tested to predict xerostomia at 24 months, the AUC reached a maximum at 0.71. This shows that, consistently with previously published articles, parotid volume variation can be used to predict xerostomia with a predictive performance which increases between week 1 and 3 but which remains inferior to what radiomics-based models yielded in this study (0.69, 0.74 and 0.86).


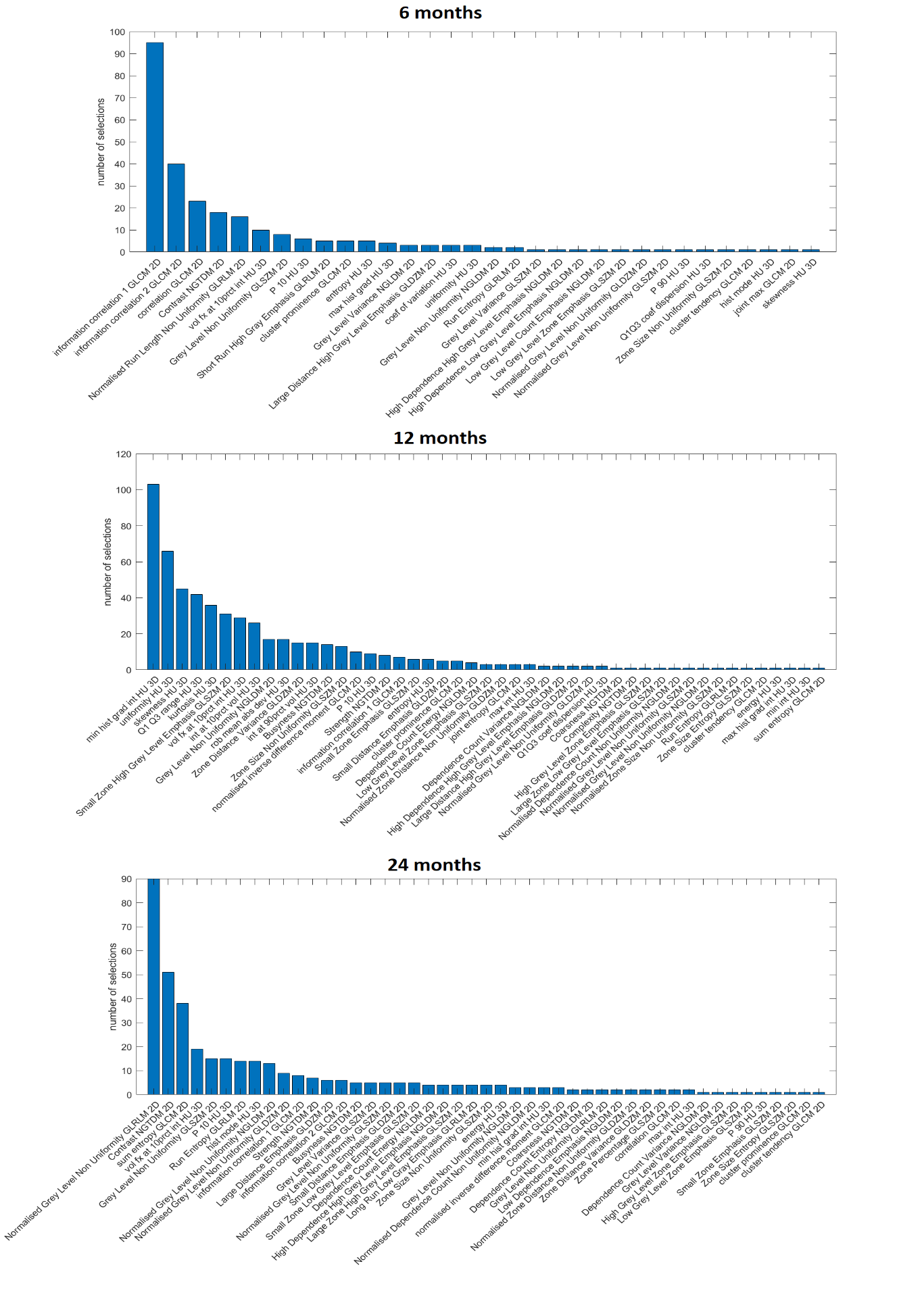
**H:** Number of selections per radiomic feature for the selected models out of 200 iterations.
